# Poor judgment of distance between nociceptive stimuli

**DOI:** 10.1016/j.cognition.2015.06.004

**Published:** 2015-10

**Authors:** Flavia Mancini, Hannah Steinitz, James Steckelmacher, Gian Domenico Iannetti, Patrick Haggard

**Affiliations:** a Department of Neuroscience, Physiology and Pharmacology, University College London, United Kingdom; b Institute of Cognitive Neuroscience, University College London, United Kingdom

**Keywords:** Somatosensory system, Pain, Nociception, Touch, Space, Spatial remapping, Parietal cortex

## Abstract

Although pain is traditionally assumed to be poorly localized, recent work indicates that spatial acuity for nociception is surprisingly high. Here we investigated whether the nervous system can also accurately estimate the distance between two nociceptive stimuli. Estimating distance implies a metric representation of spatial *relations*, a property that underlies abilities such as perceiving the size of external objects. We presented pairs of simultaneous nociceptive or non-nociceptive somatosensory stimuli, and asked participants to judge the distance between them. Judgments of distance between nociceptive stimuli were much worse than judgments of distance between non-nociceptive tactile stimuli, even on skin regions where spatial acuity for nociception exceeded spatial acuity for touch. Control experiments ruled out explanations based on inaccurate localization of double nociceptive stimuli. Thus, the nervous system poorly represents the distance between two nociceptive stimuli. The dissociation between high spatial acuity and poor distance judgment in the nociceptive system may reflect a specialization for computing accurate spatial representations useful to protect the body, rather than to perceive the size of external objects.

## Highlights

• Judgments of absolute distance between nociceptive stimuli are much worse than judgments of distance between tactile stimuli.
• Judgments of distance between two nociceptive stimuli are poor even on body regions where nociceptive spatial acuity is higher than tactile spatial acuity.
• Control experiments ruled out explanations based on inaccurate localization of double nociceptive stimuli.

## 1 Introduction

The human nervous system uses multiple representations to encode the spatial location of sensory input. The stimulus location is first mapped in a representation of the receptive surface. This initial representation is distorted, since it depends on receptive field size and density, as well as cortical magnification (Duncan & Boynton, 2007; Mancini, Haggard, Iannetti, Longo, & Sereno, 2012). At higher-order processing stages, the cortical machinery for spatial processing transforms inputs from distorted, receptor-based representations into less distorted representations based on the geometry of the external world (Azanon & Haggard, 2009; Cohen & Andersen, 2002; Maravita, Spence, & Driver, 2003). These spatial transformations allow the computation of invariant representations of external objects (Green, 1982; Taylor-Clarke, Jacobsen, & Haggard, 2004).

For instance, perceiving the distance between two somatosensory stimuli on the skin, or the size of an extended object, requires a metric representation of spatial *relations* that is unbiased by the disproportionality of early somatotopic maps (Longo, Azanon, & Haggard, 2010; Medina & Coslett, 2010). In fact, perceived tactile distance does slightly increase when moving from low to high acuity regions (Weber, 1834/1996). However, this effect is minimal when compared to the differences in innervation density of the regions involved, as estimated by tactile acuity tests (Taylor-Clarke et al., 2004). Thus, the Weber distance illusion confirms the existence of a rescaling transformation involved in tactile object perception, but suggests it is slightly underpowered.

Although the spatial processing of tactile input has been studied in depth, little is known about spatial representation of nociceptive stimuli. The traditional assumption is that pain is poorly localized. Recent findings have challenged this assumption – we have shown that spatial acuity for nociception is surprisingly high, at least in some skin regions (Mancini et al., 2013, 2014). In line with this observation, we have also found fine-grained maps of nociceptive stimuli in primary somatosensory cortex (Mancini et al., 2012). It remains unclear whether the nervous system can rescale nociceptive inputs from somatotopic representations into invariant, metric spatial representations.

We explored this question using a somatosensory distance judgment task. Specifically, we investigated whether the nervous system can accurately estimate the distance between two nociceptive stimuli. We administered pairs of simultaneous radiant heat pulses, which selectively stimulate nociceptive afferents in the skin and elicit a sensation of pinprick pain. Participants were asked to judge the absolute distance between two points. The distance between the stimuli was always higher than the spatial acuity threshold. We quantified the extent to which judged distance was proportional to the actual distance between the points. Exteroceptive sensory systems, like touch and vision, typically encode spatial properties of stimuli, such as relative position, distance and size with high precision (Haggard & Giovagnoli, 2011). Therefore, to compare tactile and nociceptive systems, participants also estimated the spatial distance between pairs of non-nociceptive mechanical stimuli, again well above the spatial acuity limit given by two-point discrimination threshold.

We conducted three experiments in 39 healthy volunteers. In Experiment 1, we stimulated the *abdomen*. In this region, spatial acuity for pain is higher than spatial acuity for touch, because of differences in peripheral innervation densities (Besne, Descombes, & Breton, 2002). In Experiment 2, we tested whether the findings of Experiment 1 could be generalized to another body part and skin type; hence, we delivered somatosensory stimuli to the glabrous skin of the hand palm. Experiment 3 was a control to evaluate whether the ability to judge distance was limited by uncertainty in localizing two simultaneous nociceptive stimuli.

## 2 Methods

### 2.1 Participants

Thirty-nine right-handed young adults took part in the study, after giving written informed consent (Experiment 1: *n* = 13, mean age ± SD, 23.3 ± 3.2, 8 females; Experiment 2: *n* = 13, mean age ± SD, 22 ± 3.3, 8 females; Experiment 3: *n* = 13, mean age ± SD, 24 ± 3.1, 7 females). The study was conducted in accordance with the principles of the Declaration of Helsinki, and was approved by the local ethics committee.

### 2.2 Nociceptive stimuli

Nociceptive stimuli were radiant heat laser pulses, which selectively activate Aδ and C nociceptors, without coactivating Aβ tactile afferents (Baumgartner, Cruccu, Iannetti, & Treede, 2005). They were generated by two identical infrared Nd:YAP lasers with a wavelength of 1.34 μm (Electronic Engineering, Florence, Italy). Note that Aδ fibers have higher thermal activation thresholds than C fibres, and there is no accepted means to stimulate Aδ fibers without coactivating lower-threshold C fibers.

Laser pulses were transmitted through optic fibers, and focused by lenses to a spot diameter of 1.3 mm. Although the laser pulse duration was 4 ms, the resulting afferent volley in the spino-thalamic system and the evoked pinprick sensation last some hundreds of milliseconds longer due to three physiological factors: (1) Delayed and long-lasting increase of temperature in the epidermis, at the depth where nociceptors are located (Leandri et al., 2006; Plaghki & Mouraux, 2003); (2) Nociceptor activation time; (3) Variable conduction velocity of primary sensory neurons, resulting in an extended spinothalamic afferent volley lasting several hundreds of milliseconds (Sikandar, Ronga, Iannetti, & Dickenson, 2013).

Laser energy (0.35–0.46 J/mm^2^) was adjusted in each subject and skin region, to elicit a clear pinprick sensation, reflecting Aδ fiber activation (Beissner et al., 2010), and achieve a mean pain intensity rating of 3 (0: no pinprick pain; 1: pinprick pain threshold; 10: worst pinprick pain imaginable). However, we allowed pain ratings to vary by ±1 score between individuals, and ±0.5 within individuals, across the explored body regions. Skin temperature of the stimulated area was monitored during every threshold measurement with an infrared thermometer, and kept at approximately 32 ± 1 °C.

### 2.3 Tactile stimuli

Somatosensory stimuli were delivered manually using two von Frey filaments (diameter 0.4 mm, weight 1 g) mounted on an electronic vernier caliper. These elicited a clear tactile percept, which was never described as painful. Stimulus duration was 1 s.

### 2.4 Experiments 1 and 2

The same procedure was applied to the hairy skin of the abdomen (dermatomes T9:11; Experiment 1), and the glabrous skin of the hand palm (median nerve territory, Experiment 2). On the abdomen, the stimulus pairs were aligned along the medio-lateral axis, so that both stimuli most likely fell within a single dermatome. On the hand palm, the stimulus pairs were aligned on the proximal-distal axis of the hand. Each experiment involved two sessions, in which either tactile or nociceptive stimuli were given. The order of sessions was counterbalanced across participants. In each session, we administered two tasks, in fixed order. First, we estimated spatial acuity thresholds, using a two-point discrimination task (2PD). Then, we assessed the ability to judge the distance between two simultaneous supra-threshold stimuli.

*Spatial acuity* was assessed using a 2PD task (Mancini et al., 2014). We randomly delivered either single stimuli (25% of trials) or two simultaneous stimuli (75% of trials). Participants reported whether they felt one or two stimuli. Importantly, we varied the intensity of the single laser pulses, so that some of them had a much higher intensity than the intensity of the two simultaneous stimuli. Therefore, the participant could not use the perceived intensity of the laser pulses as a cue for the spatial task. To measure discrimination thresholds, we used the method of limits, with interleaved ascending and descending staircases. In ascending staircases, the initial distance was 0.2 cm. In descending staircases, the initial distance was the maximal achievable for the explored body territory. The distance between the two stimuli was initially adjusted in steps of 3 cm, and then progressively reduced, until reaching the minimal distance at which the stimuli were correctly discriminated on three consecutive stimulations (Cornsweet, 1962). This distance was defined as the spatial acuity threshold. Threshold measurements were repeated three times, separately with tactile and nociceptive stimulation.

The 2PD method for evaluating spatial acuity has been criticized previously, principally because it does not control for non-spatial cues (Johnson & Phillips, 1981; Van Boven & Johnson, 1994). However, we recently demonstrated that 2PD thresholds are very similar to spatial acuity thresholds obtained with alternative methods that avoid confounds potentially present in the classical 2PD task (for example, methods based on two successive stimulations, Mancini et al., 2013, 2014). Our choice was motivated by the intention to keep the methods of the two tasks (spatial acuity and distance estimation) as similar as possible.

*Spatial distance judgment* was assessed using the following procedure. We delivered pairs of simultaneous stimuli, at four different spatial distances in randomized order. For each participant, we set four spatial distances, as 120%, 180%, 240%, and 300% of individual average spatial acuity threshold. Crucially, therefore, all the stimuli in the distance perception task were well above the 2PD threshold. The participants were asked to give absolute judgments of the distance (in cm) between the two stimuli. We showed participants a ruler with 1 cm markings prior to the experiment, to ensure familiarity with the measurement unit. If participants could not clearly detect two separate stimulus locations, they were instructed to not provide a response. However, participants always detected two separate stimuli. Four trials per spatial distance were administered in Experiment 1, and six trials per distance were administered in Experiment 2, in randomized order.

We performed the same statistical analyses in Experiments 1 and 2. We used a linear regression model to test whether the actual distance between the two stimuli linearly predicted the judged distance. We separately modeled the relation between actual and judged distances, for each individual participant and modality (nociception, touch). Both actual and judged distances were expressed as a proportion of their respective maximum values. At individual level, we used simple regression to test for a linear relation between perceived and actual distance. At the group level, we compared the slopes (*β* estimates) and coefficients of determination (*R*^2^, a measure of the goodness of fit of the model) for the linear models for touch and nociception. We performed a paired *t*-test comparing *β* estimates for touch and nociception linear functions, and we estimated the standardized effect size (Cohen’s *d*) of this difference, as follows: *d* = *t*/√*N*. Finally, we compared *R*^2^ coefficients for touch and nociception linear functions using a non-parametric, related-samples Wilcoxon signed rank test, because the distribution of *R*^2^ coefficients was not normal. We estimated the effect size of this comparison as follows: ES = *z*/√*N*.

## 3 Experiment 3

This experiment investigated whether performance in judging the distance between two simultaneous nociceptive stimuli depended on uncertainty in localising double stimulations. Put simply, nociceptive distance judgments might be poor if the simultaneous co-occurrence of stimulation at two sites strongly impaired the ability to judge location. Participants sat in front of a computer screen, and vision of the right hand was occluded. As in Experiment 2, nociceptive stimuli were delivered to the palm of the right hand. We first evaluated spatial acuity, with the same procedure used in Experiments 1 and 2. We then set four spatial distances, at 120%, 180%, 240%, and 300% of individual average spatial acuity threshold. The experiment was as follows. We delivered 6 blocks of 8 randomized trials: on 4 trials in each block, a single radiant heat pulse was administered at random palm locations; on the other 4 trials of the block, pairs of simultaneous pulses (one trial per spatial distance) were delivered, aligned along the proximal-distal axis of the hand palm. On each trial, a picture of the stimulus location was taken by a webcam located above the hand. After the stimulation, participants were first asked to report whether one or two points were detected. If they detected two stimuli, they were asked to verbally judge the absolute distance, in cm, between the two stimuli. Then, an image of the palm of the hand (approximately at real size) appeared on a computer screen in front of the participant. Participants were asked to click in the position corresponding to each nociceptive stimulus location. Thus, on the same trial we measured both distance judgment and localization.

To place the actual and judged locations into a common coordinate frame, we used the two-point registration method developed by Bookstein (Bookstein coordinates; Bookstein, 1991). We defined two reference points on the hand as being points (0, 0) and (1, 0): specifically, the midpoint between the base of the middle and ring fingers was set as point (0, 0), and the center of the wrist line as point (1, 0). For each trial, *x* and *y* pixel coordinates of the stimulus locations from the webcam picture were transformed into coordinates centered on the *x* and *y* pixel coordinates of the two hand references, from the same picture. Furthermore, the *x* and *y* coordinates of the judged locations, from the clicks on the hand image, were similarly transformed into coordinates centered on the two references of the hand image.

This procedure has important benefits (Longo & Haggard, 2010; Mancini, Longo, Iannetti, & Haggard, 2011). First, it places the locations of the stimuli (coded from a photograph of each participant’s hand) and the locations of the responses (defined by mouse clicks) into a common body-scaled, reference frame, allowing meaningful comparisons. Second, it defines unit length relative to the size of each participant’s hand, removing individual differences in overall hand size, thus allowing cross-participants averaging.

We then calculated the Variable Error (VE) of localization, which represents the precision, or uncertainty, of localization. The VE is defined as the standard deviation of a set of responses from the average response location. We averaged VE of localization across the proximal-distal and ulnar-radial components. We then performed three planned comparisons: (a) between VE for a single stimulus and the average VE for each of the stimuli in the double simultaneous stimulation condition, (b) between VE for a single stimulus and the VE for the most proximal stimulus in the double simultaneous stimulation condition, and (c) between VE for a single stimulus and the VE for the most distal stimulus in the double simultaneous stimulation condition. The standardized effect size (Cohen’s *d*) for each paired comparison was calculated as follows: *d*_VE_ = *t*/√*N*.

As in Experiments 1 and 2, we tested whether there was a significant linear relation between actual and judged distance between two simultaneous nociceptive stimuli. Note that participants first judged the distance between the stimuli, then localised the sensation on a visual image of the hand. At the single subject level, the statistical analyses were identical to Experiments 1 and 2. At group level, we compared the *β* estimates of the linear functions describing the relation between actual and judged distance against unity (i.e., *β* = 1), using a one-sample *t*-test.

Finally, we addressed the key question of whether the poor performance in judging the distance between two nociceptive stimuli simply reflects uncertainty in stimulus localisation. To test this hypothesis, we compared the effect size for the effect of number of stimuli on precision of localisation (*d*_VE_) to the effect size for the impairment in nociceptive distance judgement (*d*_β_). As the effect sizes are both standardised, they may be compared directly despite involving different tasks. We calculated the difference [*d*_β_ − *d*_VE_], and the variance (*V*) of this difference as follows: *V*_diff_ = *V*_β_ + *V*_VE_ − 2*r*√*V*_β_ √*V*_VE_ (Borenstein, Hedges, Higgins, & Rothstein, 2009), where *r* is the coefficient of correlation between the two effects. We used the variance estimate to calculate a 95% confidence interval for this difference: ±2.179 ∗ SE_diff_.

## 4 Results

### 4.1 Experiments 1 and 2

#### 4.1.1 Spatial acuity

**Fig. 1:**
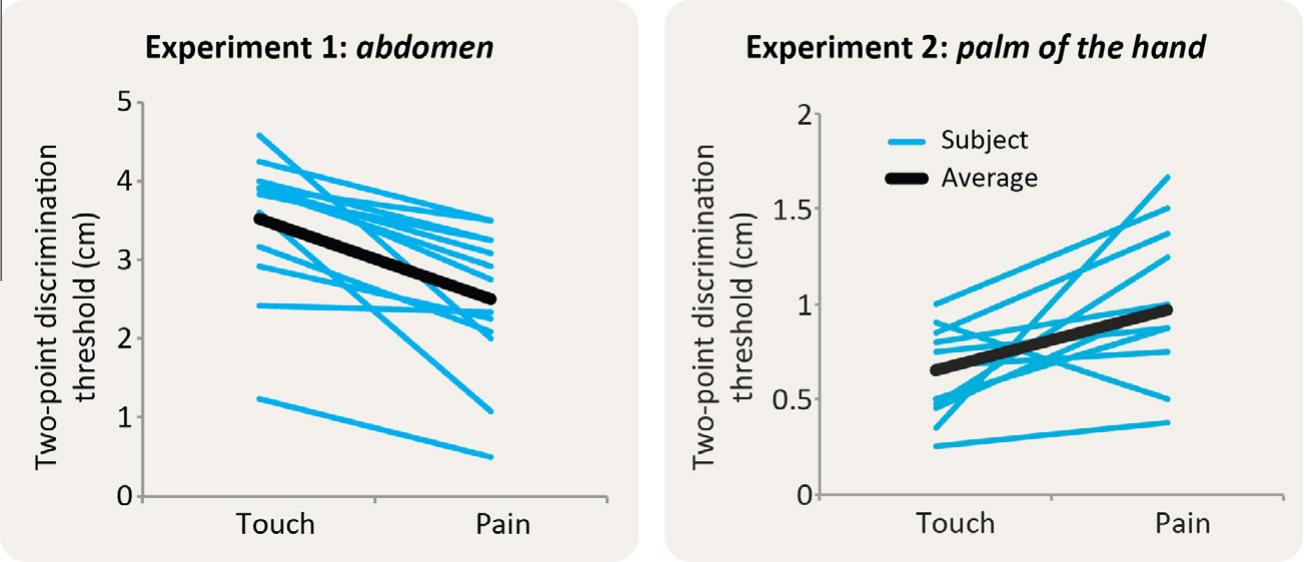
Mean two-point discrimination (2PD) thresholds for nociception and touch on the abdomen (Experiment 1, *n* = 13, left panel) and on the palm of the hand (Experiment 2, *n* = 13, right panel). Thin lines represent individual participants, while thick lines depict the group averages.

In every participant tested in Experiment 1, 2PD thresholds for nociception were lower than 2PD thresholds for touch on the abdomen (paired *t*-test: *t*_12_ = −4.98, *p* < 0.0001; Fig. 1, left panel). This is one of the few body regions in which nociception shows higher spatial acuity than touch (Mancini et al., 2014), and is in line with evidence of high density of free nerve endings (Besne et al., 2002). In contrast, on the glabrous skin of the hand palm (Experiment 2), 2PD thresholds for nociception were higher than 2PD thresholds for touch on the palm of the hand (paired *t*-test: *t*_12_ = −2.49, *p* = 0.028; Fig. 1, right panel), as previously reported (Mancini et al., 2014).

#### 4.1.2 Judgment of spatial distance

##### 4.1.2.1 Experiment 1: hairy skin (abdomen)

**Fig. 2:**
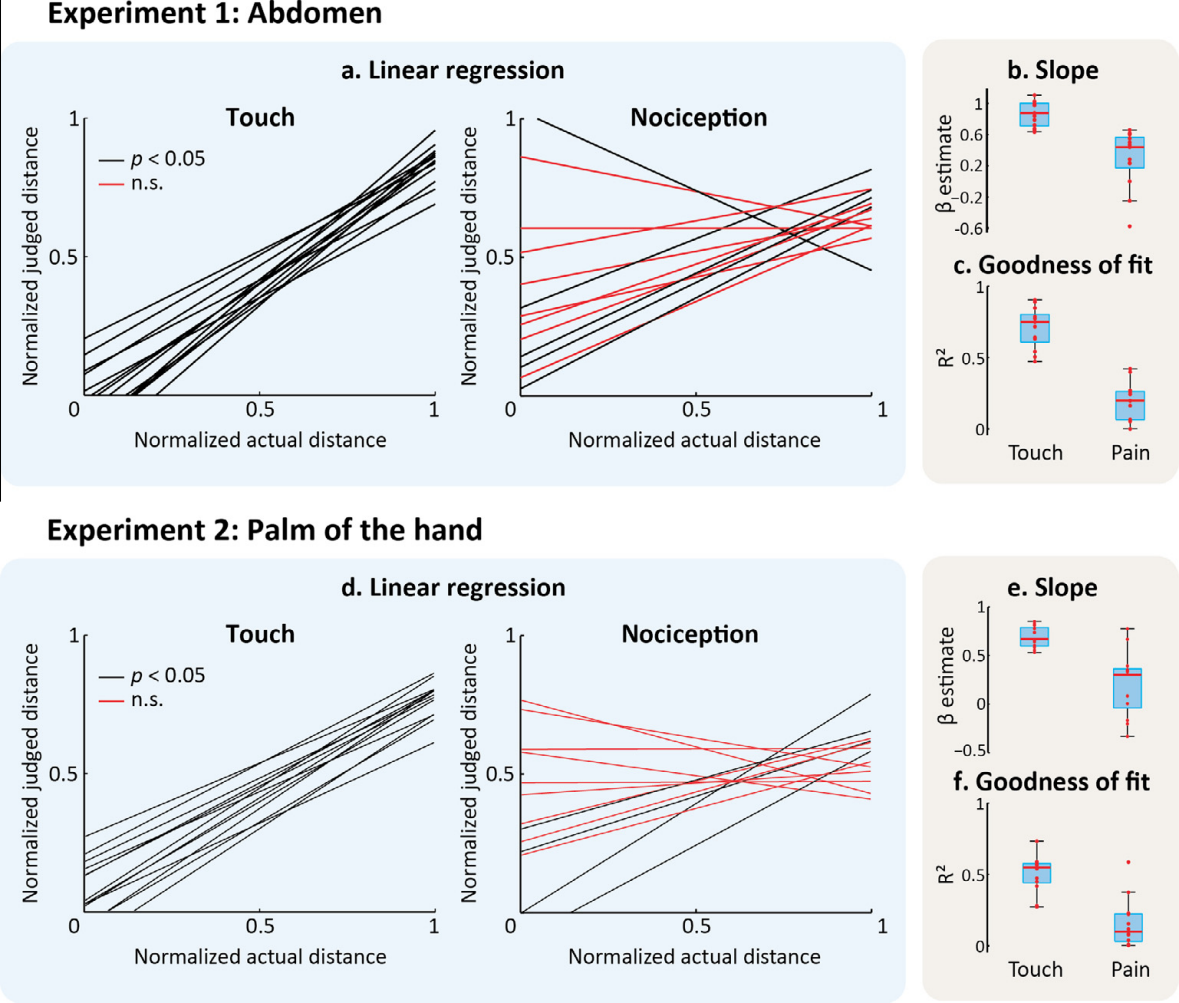
Experiment 1: abdomen. (a) *Regression lines* for the relation between normalized actual and judged distance between pairs of tactile and nociceptive stimuli. Each line depicts the fit of an individual participant data. Black lines represent significant regression models (*p* < 0.05), while red lines depict non-significant models. (b) *Slope*: Box plots for the *β* estimates derived from the regression models for touch and nociception. On each box, the central mark is the median, the edges of the box are the 25th and 75th percentiles, the whiskers extend to the most extreme data points not considered outliers, and outliers are plotted individually. The *β* values of each individual participant are represented with dots. (c) *Goodness of fit*: Box plots for the coefficients of determination (*R*^2^) derived from the regression models for touch and nociception. The *R*^2^ coefficients of each individual participant are represented with dots. Experiment 2: Palm of the hand. (d) *Regression lines* for the relation between normalized actual and judged distance between pairs of tactile and nociceptive stimuli. Each line depicts the fit of an individual participant data. Black lines represent significant regression models (*p* < 0.05), while red lines depict non-significant models. (e) *Slope*: Box plots for the *β* estimates derived from the regression models for touch and nociception. On each box, the central mark is the median, the edges of the box are the 25th and 75th percentiles, the whiskers extend to the most extreme data points not considered outliers, and outliers are plotted individually. The *β* values of each individual participant are represented with dots. (f) *Goodness of fit*: Box plots for the coefficients of determination (*R*^2^) derived from the regression models for touch and nociception. The *R*^2^ coefficients of each individual participant are represented with dots.

The actual distance between two simultaneous touches on the abdomen strongly predicted tactile distance judgments in a linear fashion (Figs. 2a and S1). Indeed, the linear model was significant in every individual participant tested (Table 1). In contrast, participants were markedly poorer at judging the distance between two nociceptive stimuli. The linear regression models for nociception were significant in only five of the 13 participants tested (Table 1, Figs. 2a, and S1), despite the relatively high nociceptive spatial acuity on the abdomen (Fig. 1, left panel). More importantly, both the slopes and the coefficients of determination from individual linear regressions were lower for nociception than for touch (Fig. 2b and c; *β* estimates: *t*_12_ = 4.97, *p* < 0.0001, *d* = 1.38; *R*^2^: Wilcoxon signed rank test, *p* = 0.001, ES = 0.88). A visual inspection of the raw data (Fig. S1) and of the residuals of the regression model did not highlight any consistent non-linear relation between actual and judged distance of pairs of nociceptive stimuli.

##### 4.1.2.2 Experiment 2: glabrous skin (hand palm)

The results from a second group of participants tested on the glabrous skin confirmed the observations of Experiment 1. Again, the relationship between actual and judged distance was strongly linear for touch, but not for nociception (Figs. 2d and S2). For touch, the linear regression models of this relationship were significant in each participant. However, for nociception, they were significant only in four of the 13 tested volunteers (Table 2, Figs. 2d, and S2). Again, the slopes and the coefficients of determinations derived from individual linear regressions were lower for nociception than for touch (Fig. 2e and f; *β* estimates: *t*_12_ = 4.83, *p* < 0.001, *d* = 1.34; *R*^2^: Wilcoxon signed rank test, *p* = 0.004, ES = 0.8).

### 4.2 Experiment 3

**Fig. 3:**
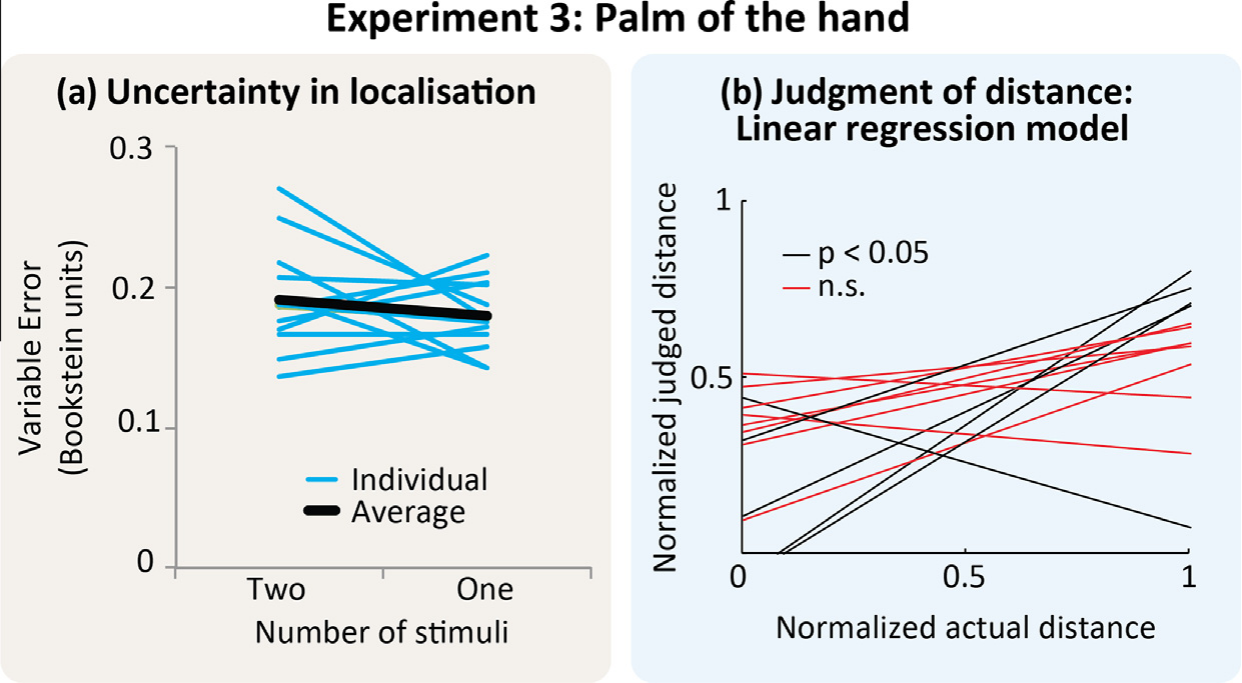
Experiment 3: Palm of the hand. (a) *Variable Error* in localization as a function of number of nociceptive stimuli. Thin lines represent individual participants, while the thick line depicts the group average. (b) *Regression lines* for the relation between normalized actual and judged distance between pairs of nociceptive stimuli. Each line depicts the fit of an individual participant data. Black lines represent significant regression models (*p* < 0.05), while red lines depict non-significant models.

In line with Experiments 1 and 2, the judgments of distance between two simultaneous nociceptive stimuli were rather poor. There was a significant positive linear relation between actual and judged distance (in cm) only in 4 of 13 participants (Fig. 3b and Table 3). The comparison between *β* estimates and unity (*β* = 1) was also highly significant (*t*_12_ = −7.366, *p* < 0.0001, *d*_β_ = 2.04).

However, the Variable Error of nociceptive localization was comparable for a single nociceptive stimulus and for two simultaneous stimulations (Fig. 3a; *t*_12_ = 0.98, *p* = 0.346, *d*_VE_ = 0.27). VE for single stimulation did not differ from VE for the more distal of the double simultaneous stimuli, (*t*_12_ = 1.03, *p* = 0.322, *d* = 0.29) nor from VE for the more proximal of the double simultaneous stimuli (*t*_12_ = 0.88, *p* = 0.394, *d* = 0.24).

Finally, we tested whether the impairment in nociceptive distance judgement exceeded the difference in precision of localising two nociceptive stimuli vs. one point. We did this by comparing the effect size for the difference between the distance judgement regression slope and unity (*d*_β_ = 2.04), with the effect size for the increase in variable error from single to double simultaneous stimulation (*d*_VE_ = 0.27). The null hypothesis for this comparison (*d*_β_ = *d*_VE_) states that poor nociceptive distance judgement merely reflects difficulty in localising double simultaneous stimulation. Were this the case, the suboptimal performance in distance judgement could be explained by the drop in precision of nociceptive localisation for double stimuli. Crucially, the 95% confidence interval around the difference did not include 0 (1.54–2). We thus rejected the null hypothesis, and conclude that the poor metric representation of nociceptive distance is not explained by uncertainty in localization for double simultaneous stimulation.

## 5 Discussion

This study indicates that judgments of the spatial distance between two simultaneous nociceptive stimuli were much poorer than judgments of comparable distances between tactile stimuli. In 27 of the 39 participants tested in three experiments the relation between actual and judged distance between two nociceptive stimuli was *not* positively linear (Figs. 2 and 3). A visual inspection of the data did not reveal any consistent non-linear relation between actual and judged distance across individuals (Figs. S1 and S2).

Importantly, poor estimation of nociceptive spatial distance cannot simply be explained in terms of low spatial resolution of the nociceptive system. Spatial acuity for nociception, as measured by a 2PD task, was high in the body regions we studied. In the case of the hairy skin of the abdomen, spatial acuity for nociception was even higher than spatial acuity for touch (Experiment 1; Fig. 1), due to the dense innervation of nociceptive free nerve endings (Besne et al., 2002). Moreover, the distance between the two stimuli was specifically adjusted to the spatial acuity threshold for each individual, somatosensory modality, and skin region. The distances judged were *always* above the acuity threshold for the specific individual’s tactile or nociceptive system. Lastly, participants always detected two stimuli: this rules out the possibility that distance judgment for pain is poor because of a simple sensory fusion, or funneling, leading to nearby peaks of cortical activity being combined (Bekesy, 1958; Chen, Friedman, & Roe, 2003; Sherrick, 1964).

An additional feature of our results seems to rule out funneling. Funneling-type mechanisms should produce perceptual compression of shorter distances (Gardner & Spencer, 1972). This would tend to increase the slope of the relation between judged and actual distance over the whole range. In fact, we observed the opposite effect: these slopes were lower for nociceptive than for tactile distances. Therefore, funneling cannot readily explain our results.

Furthermore, Experiment 3 showed that poor estimation of nociceptive distance could not be explained by the increase in uncertainty about localization when two nociceptive stimuli are delivered simultaneously, compared to a single stimulus alone. Specifically, we showed comparable variable error of localisation for single and double simultaneous nociceptive stimuli. The effect size for impairment in distance judgment was also significantly larger than the effect size for uncertainty in localisation under double stimulation.

Given that spatial acuity and judgment of distance appear to be dissociated in the nociceptive system, they presumably involve different neural substrates.

Spatial acuity reflects the receptive field (RF) size and density of skin receptors, as well as cortical magnification in primary somatosensory cortex (SI) (Duncan & Boynton, 2007; Mountcastle, 2005). Because the somatotopic representation of the receptor surface in SI is highly distorted, it cannot provide the invariant geometric representation of space that characterizes our perception of the external objects.

For instance, consider the cortical activity evoked by two stimuli, at a given distance apart, presented to a poorly innervated skin region, or to a highly innervated skin region. In a disproportionate SI map, the separation between the evoked activities for the two points will be lower for the poorly innervated skin region than for the highly innervated skin region. However, to recognize the stimulus object as the same in these two cases, the brain would need to compute an *invariant* representation of distance. The distance between the two loci of neural activity in the SI map would need to be rescaled into a spatial metric system. To be truly invariant, this spatial representation should be independent on RF size and density (Taylor-Clarke et al., 2004).

These rescaling transformations are computed by several regions in the posterior parietal cortex (Azanon & Haggard, 2009; Cohen & Andersen, 2002; Longo et al., 2010; Maravita et al., 2003). In particular, the angular gyrus is involved in the estimation of tactile spatial distances (Spitoni, Galati, Antonucci, Haggard, & Pizzamiglio, 2010; Spitoni et al., 2013). The angular gyrus is also thought to play a role in the representation of magnitude (Walsh, 2003), which may be closely linked to the representation of spatial extent, or distance. Little is known of how nociceptive inputs are coded in the posterior parietal cortex, though dense connections between central-opercular regions and posterior parietal regions are known to exist (Eickhoff et al., 2010; Jones, Coulter, & Hendry, 1978; Uddin et al., 2010).

How, then, is spatial information coded in the nociceptive system? We emphasize that nociception does involve spatial processing. Several lines of evidence suggest that both spatiotopic and somatotopic factors play an important role in pain perception. In particular, the position in egocentric space of a stimulated body part modulates pain intensity and the cortical response to a nociceptive stimulus (Gallace, Torta, Moseley, & Iannetti, 2011; Sambo et al., 2013). Thus, a single nociceptive stimulus can be remapped in external frames of references. However, our results suggest that nociception has little access to *metric* representations coding the position of one input relative to another.

The dissociation between high spatial acuity and poor distance judgment of nociceptive stimuli may have a functional significance. The functional role of pain is to signal danger and potential tissue damage (Craig, 2003; Downar, Crawley, Mikulis, & Davis, 2002; Legrain, Iannetti, Plaghki, & Mouraux, 2011; Moseley, Gallace, & Spence, 2012). Information about the location of nociceptive stimuli is important for organizing functional defensive or orienting responses such as withdrawal. However, information about the geometric properties of external stimuli, such as spatial distance, is less important for survival and is likely to rely more on mechanosensation rather than nociception.

## Figures and Tables

**Table 1:** *Experiment 1:* Significance of the linear regression model of the relation between actual and judged distance, in each individual participant. Tactile and nociceptive stimuli were given to the hairy skin of the abdomen. Highlighted in italics are *p*-values > 0.05.

Participant	Touch		Pain	
	*F*	*p*	*F*	*p*
1	47.98	<0.0001	0.94	*0.348*
2	12.50	0.003	0.97	*0.341*
3	14.26	0.002	0.79	*0.389*
4	47.38	<0.0001	4.97	0.043
5	51.26	<0.0001	4.47	*0.053*
6	131.47	<0.0001	4.85	0.045
7	25.00	<0.0001	9.28	0.009
8	41.62	<0.0001	3.44	*0.085*
9	16.54	0.001	10.14	0.007
10	35.23	<0.0001	<0.0001	*1.000*
11	23.78	<0.0001	0.99	*0.337*
12	112.04	<0.0001	5.16	0.039
13	77.72	<0.0001	2.73	*0.121*

**Table 2:** *Experiment 2:* Significance of the linear regression model of the relation between actual and judged distance, in each individual participant. Tactile and nociceptive stimuli were given to the glabrous skin of the hand palm. Highlighted in italics are *p*-values > 0.05.

Participant	Touch		Pain	
	*F*	*p*	*F*	*p*
1	30.29	<0.0001	3.89	*0.061*
2	19.29	<0.0001	2.82	*0.107*
3	26.11	<0.0001	0.00	*0.987*
4	25.14	<0.0001	6.36	0.019
5	8.44	0.008	30.23	<0.0001
6	58.05	<0.0001	0.81	*0.377*
7	30.61	<0.0001	<0.001	*0.985*
8	28.07	<0.0001	1.85	*0.187*
9	28.78	<0.0001	12.92	0.002
10	8.05	0.010	2.327	*0.141*
11	17.52	<0.0001	6.11	0.022
12	28.20	<0.0001	0.16	*0.692*
13	15.49	0.001	1.77	*0.197*

**Table 3:** *Experiment 3:* Significance of the linear regression model of the relation between actual and judged distance, in each individual participant. Tactile and nociceptive stimuli were given to the glabrous skin of the hand palm. Highlighted in italics are *p*-values > 0.05.

Participant	*F*	*p*
1	34.26	<*0.001*
2	0.22	*0.644*
3	5.38	*0.033*
4	3.39	*0.080*
5	0.09	*0.764*
6	3.56	*0.075*
7	0.24	*0.628*
8	6.84	0.015
9	12.69	0.002
10	1.45	*0.245*
11	11.34	0.003
12	1.43	*0.246*
13	2.30	*0.144*
