# Supplementary material for: Poor judgment of distance between nociceptive stimuli
Source: Cognition. 2015 Oct;143:41–7. doi: 10.1016/j.cognition.2015.06.004 (PMC4534310; doi:10.1016/j.cognition.2015.06.004)
Supplement: Supplementary data 1 [file mmc1.pdf]

Supplementary Figures

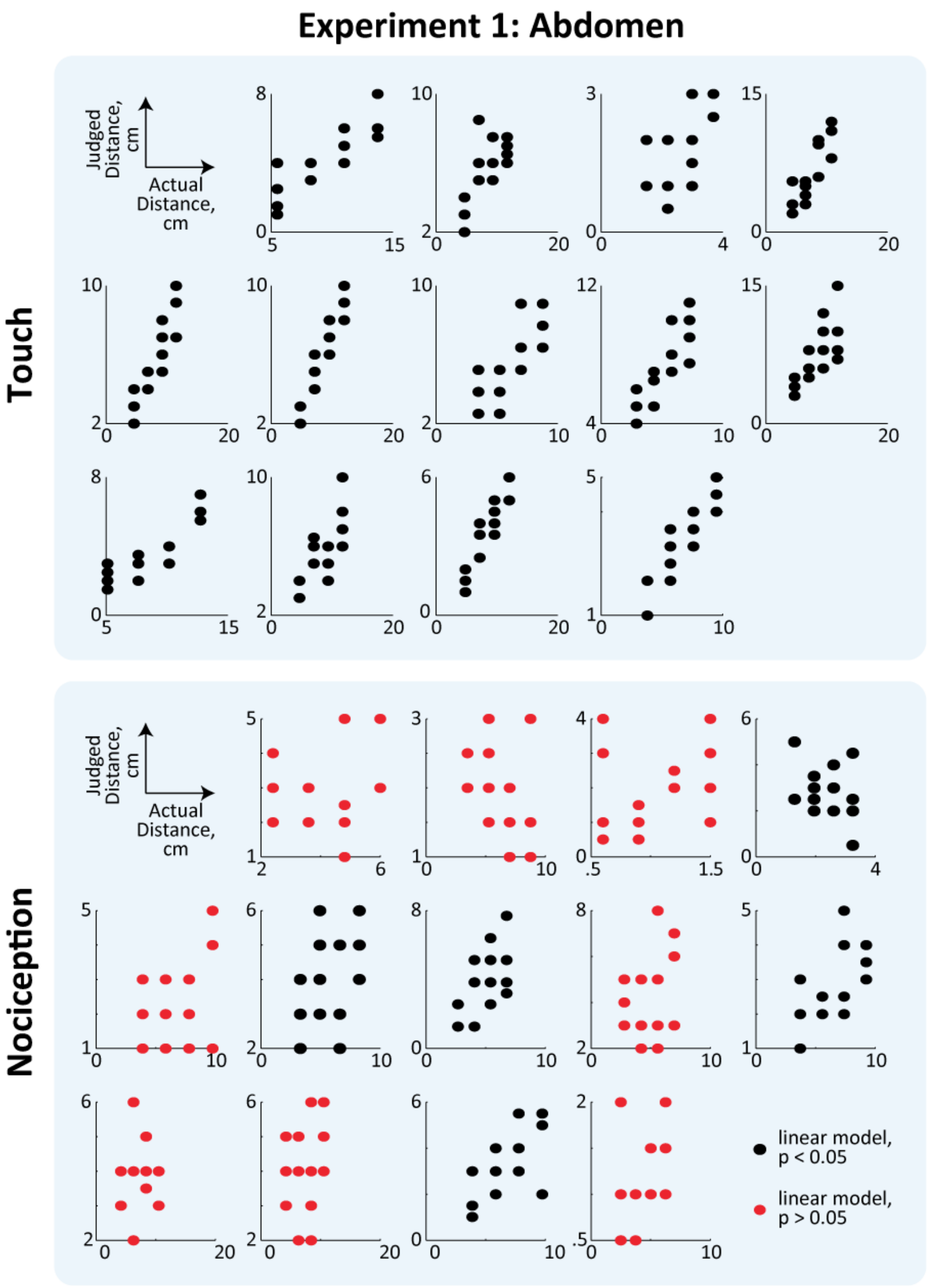

Figure S1. Experiment 1 (abdomen): raw data.
Each plot represents the actual distance (cm, x-axis) by judged distance between simultaneous stimuli (cm, y-axis), for each participant and stimulus modality. Note that the x-axes differ in each participant because the actual distance was scaled on the individual spatial acuity threshold (120:300% above 2PD threshold). Black markers indicate significant linear regression models ($p < 0.05$), while red markers indicate non-significant linear models ($p > 0.5$).

Experiment 2: Hand palm

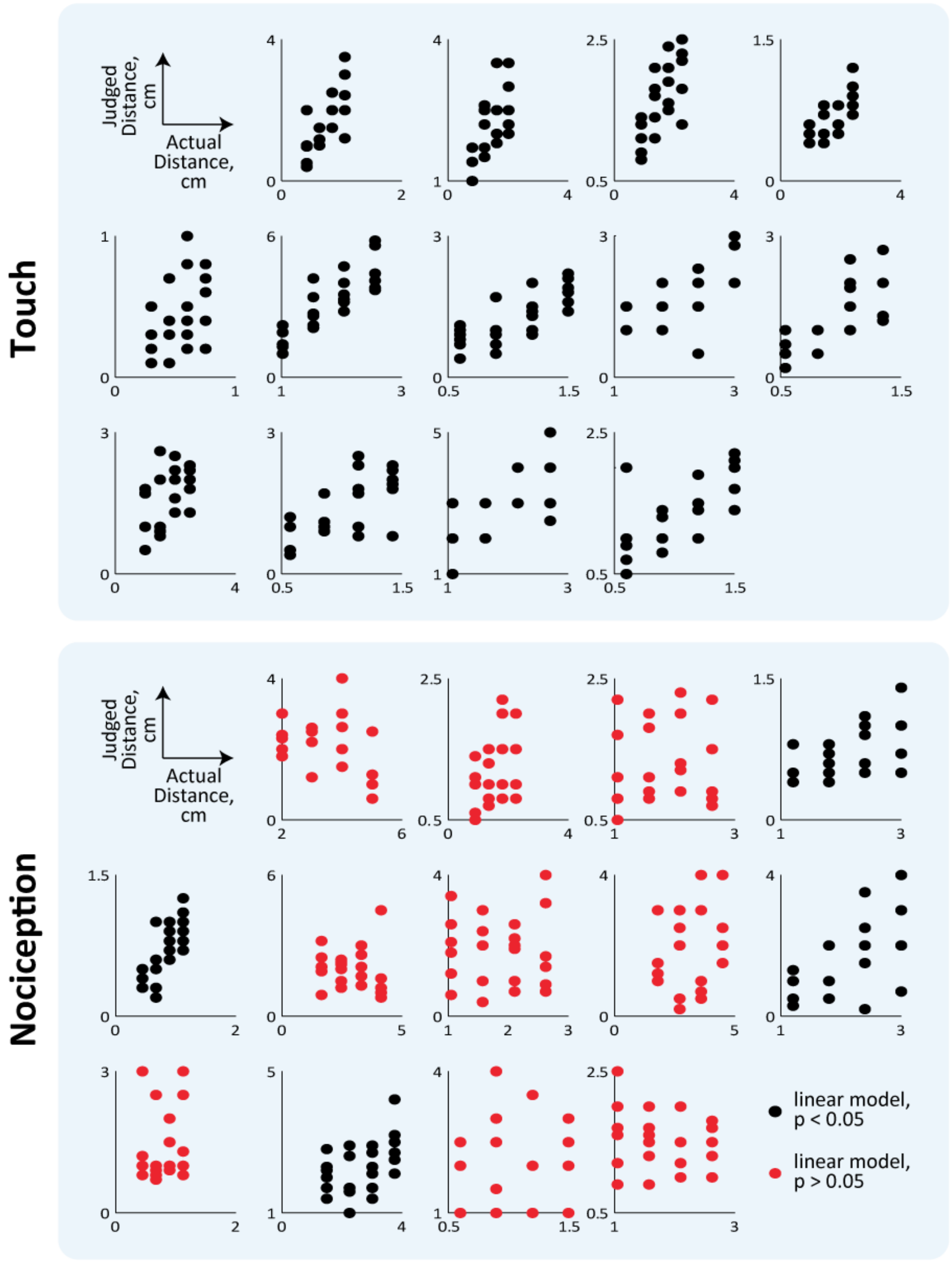

Figure S2. Experiment 1 (hand palm): raw data.
Each plot represents the actual distance (cm, x-axis) by judged distance between simultaneous stimuli (cm, y-axis) for each participant, and stimulus modality. Note that the x-axes differ in each participant because the actual distance was scaled on the individual spatial acuity threshold (120:300% above 2PD threshold). Black markers indicate significant linear regression models ($p < 0.05$), while red markers indicate non-significant linear models ($p > 0.5$).
